# Chromatin accessibility associates with protein-RNA correlation in human cancer

**DOI:** 10.1038/s41467-021-25872-1

**Published:** 2021-09-30

**Authors:** Akshay Sanghi, Joshua J. Gruber, Ahmed Metwally, Lihua Jiang, Warren Reynolds, John Sunwoo, Lisa Orloff, Howard Y. Chang, Maya Kasowski, Michael P. Snyder

**Affiliations:** 1 Department of Genetics, Stanford University, Stanford, CA USA; 2 Division of Oncology, Department of Medicine, Stanford University School of Medicine, Stanford, CA USA; 3 Center for Personal Dynamic Regulomes and HHMI, Stanford University, Stanford, USA; 4 Division of Head and Neck Surgery, Department of Otolaryngology, Stanford University School of Medicine, Stanford, CA USA; 5 Department of Pathology, Stanford University School of Medicine, Stanford, CA USA; 6 Division of Pulmonary and Critical Care Medicine, Department of Medicine, Stanford University School of Medicine, Stanford, CA USA; 7 Sean N. Parker Center for Allergy and Asthma Research at Stanford University, Stanford University, Stanford, CA USA

**Keywords:** Gene regulatory networks, Epigenomics, Cancer genomics

## Abstract

Although alterations in chromatin structure are known to exist in tumors, how these alterations relate to molecular phenotypes in cancer remains to be demonstrated. Multi-omics profiling of human tumors can provide insight into how alterations in chromatin structure are propagated through the pathway of gene expression to result in malignant protein expression. We applied multi-omics profiling of chromatin accessibility, RNA abundance, and protein abundance to 36 human thyroid cancer primary tumors, metastases, and patient-match normal tissue. Through quantification of chromatin accessibility associated with active transcription units and global protein expression, we identify a local chromatin structure that is highly correlated with coordinated RNA and protein expression. In particular, we identify enhancers located within gene-bodies as predictive of correlated RNA and protein expression, that is independent of overall transcriptional activity. To demonstrate the generalizability of these findings we also identify similar results in an independent cohort of human breast cancers. Taken together, these analyses suggest that local enhancers, rather than distal enhancers, are likely most predictive of cancer gene expression phenotypes. This allows for identification of potential targets for cancer therapeutic approaches and reinforces the utility of multi-omics profiling as a methodology to understand human disease.

## Abstract (web summary)

Studies show the cancer transcriptome correlates poorly with the cancer proteome, questioning the role of chromatin regulation. Here the authors demonstrate proximal-gene-body chromatin elements and transcription predict abundances of differentially expressed proteins in thyroid and breast cancers.

## Introduction

The central dogma describes that DNA sequences code for RNA, and RNA is translated to produce protein. However, additional layers of regulation occur at the level of chromatin, as well as at the levels of post-transcriptional and post-translational control. The general degree to which these levels are tuned during health and human disease remains to be fully explored^1–3^.

Chromatin accessibility is a hallmark of active enhancers, which are *cis* regulatory elements of gene expression^4^. Chromatin accessibility and RNA abundance are often measured together to map the regulatory context of gene expression, excluding measurements of the proteome. Although RNA is used as surrogate marker for protein expression, many studies have shown that RNA and protein levels are markedly different (~correlation = 0.3)^5,6^. It remains to be determined whether the chromatin context associates with the cancer proteome.

Cancer reprogramming confers drastic changes in RNA and protein levels^6,7^ and the chromatin landscape^8,9^. Cells reprogram their identity to express growth, migratory, and immune evasion pathways. Two interesting reprogramming situations occur in solid tumors: (1) progression from normal tissue to primary tumor and then metastatic tumor; (2) divergence of primary tumors into molecular subtypes that develop from the same original tissue type^10,11^. In both situations, the tissue of origin transforms its cellular identity, which can be distinguished by its molecular features^12,13^. However, it is unclear how dynamic chromatin landscapes relate to the dynamic protein expression in cancer.

We conducted a multi-omics investigation of how cancer alters the chromatin accessibility landscape to express different proteins and furthermore provide a framework to prioritize genes that define pathological stages and molecular cancer subtypes^14^. We studied tumor progression using a cohort of metastatic thyroid carcinoma, which includes 36 patients’ tissue samples of patient-matched normal thyroid, primary tumor, and metastatic samples. Our integrative analytical framework unexpectedly identifies regulatory elements in the gene body that associate with differential transcription and translation. We find that proximal enhancer sites, rather than upstream distal enhancers, have preferential activity in driving changes in protein abundance that are likely most important in establishing the malignant state. Proteins under coordinated gene regulation likely control the cancer phenotype and thus they could serve as better targets for diagnostics and therapeutics.

## Results

### Curation of clinical samples, data acquisition, and primary analysis

**Fig. 1:**
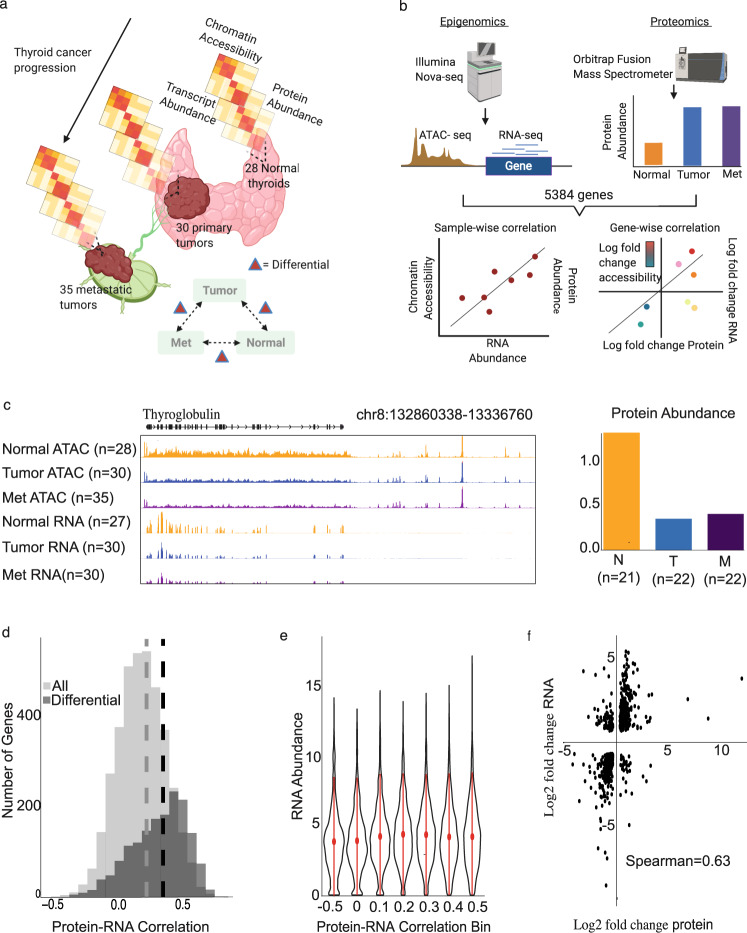
Integrative multi-omic analysis of thyroid cancer cohort. **a** Cohort description, 93 samples of patient-matched samples of lymph-node metastases, primary thyroid tumors, and adjacent normal thyroid were assayed. Three omics are integrated to profile chromatin accessibility, transcript abundances, and protein abundance. **b** Analytical framework, assay for transposase-accessible chromatin using sequencing (ATAC-seq), RNA-seq, and proteomics resulted in measurements for 5384 genes, which were used in correlation analyses across omics. **c** Visualization of the omic data for thyroglobulin (*TG*). Biologically independent tissue chunks from each disease stage (i.e., normal, tumor, and met) are averaged to produce the signal plots. **d** Distribution of protein–RNA correlation comparing all genes vs differentially expressed genes. **e** Distribution of RNA abundance for bins of protein–RNA correlations. **f** Gene-wise correlation of differentially expressed genes at the RNA level and differentially expressed proteins across all pairwise comparisons.

To investigate the gene expression and other molecular alterations that are induced in the progression from normal thyroid to primary tumor and metastases, we analyzed clinical specimens of patient-matched metastases, primary tumor, and normal thyroid tissue. We collected 30 primary tumors (Tumor), 28 patient-matched normal thyroids that were taken contralateral to tumor (Normal), and 35 local lymph node metastases (Met) (Fig. 1a). Fresh-frozen tissues were aliquoted to collect nuclei, RNA, and proteins for the downstream multi-omics assays. This allowed us to identify how chromatin structure relates to the abundance of transcripts and proteins in thyroid cancer. To interrogate the chromatin landscape, we conducted assay for transposase-accessible chromatin using sequencing (ATAC-seq), which uses a transposase to selectively integrate sequencing adapters into accessible chromatin^15^. RNA abundances were quantified by sequencing ribosomal RNA-depleted RNA^16^. Protein abundance was quantified by TMT-labeled shotgun proteomics with data-dependent acquisition^17^ (see “Methods”).

In cancer, we expect a subset of genes’ expression levels and associated chromatin accessibility to change in the progression of thyroid cancer^8^. Thus, we conduct differential testing at each omic level to determine which genes and chromatin features are dynamic in the progression of thyroid cancer (Fig. 1a). We define a differentially expressed gene if it is significantly differential (false discovery rate (FDR) < 0.1) between any of the pairwise comparisons (i.e., Tumor vs Normal, Met vs Normal, or Met vs Tumor) at either the protein or RNA level. Furthermore, we quantify differential chromatin accessibility for regions that are predicted to regulate gene expression.

Our analytical framework (Fig. 1b) aims at measuring molecules from the flow of information from chromatin to protein. We identified 5384 proteins, which are detected at the RNA level and have predicted chromatin features that regulate expression. Fifty-four samples were profiled for chromatin accessibility, RNA abundance, and protein abundance (18 Normal tissues, 18 Tumors, and 18 Mets). We conducted two types of correlation comparisons (1) sample-wise correlation and (2) gene-wise correlation^5^. In sample-wise correlation, following the flow of information for each gene, we compare two correlation values: correlation between chromatin accessibility at a regulatory element and RNA abundance, and correlation between RNA abundance and protein abundance. In gene-wise correlation, we test correlation of fold changes of differential accessibility, differential RNA abundances, and differential protein abundances for all pairwise comparisons. Pairwise comparisons produce similar results to a standard analysis of variance test but gives additional information of dynamic changes between pairs. Our analysis shows that overall tumors and metastases form a cluster that is distinct from the normal thyroid samples at the RNA and protein levels (Supplementary Fig. 1)

Chromatin accessibility and RNA abundance are mapped to the genome as shown on an example genome track (Fig. 1c). The signal tracks an average across many samples (28 Normal ATAC, 30 Tumor ATAC, 35 Met ATAC, 27 Normal RNA, 30 Tumor RNA, and 30 Met RNA). Since proteomics quantifies relative abundance of fragmented peptides, it measures abundance at the protein level. Thus, we visualize this as bar graphs with bars summarizing relative protein abundance in each tissue type (Fig. 1c). These signals are also averaged across samples (21 Normals, 22 Tumors, and 22 Mets). In the example of the thyroglobulin gene, shown here, the chromatin accessibility surrounding the transcription start site (TSS) of thyroglobulin follows a positive correlation with RNA abundance and protein abundance in averaged levels in normal thyroids, tumors, and metastases (Fig. 1c).

We next examined whether there is a difference in protein–RNA correlation between all detected proteins to differentially expressed RNAs for all pairwise comparisons. The distribution of protein–RNA correlation across all genes is centered at 0.22, and 85% of genes have a positive correlation (Fig. 1d), and the tissue type does not affect this correlation (Supplementary Fig. 1e). Among the detected proteins, 1049 genes at the protein level and 943 genes at the RNA level were significantly differential for all pairwise comparisons. Analyzing only the genes that are significantly differential by either measure of expression, the distribution of protein–RNA correlation significantly shifted toward higher correlation with a median spearman correlation of 0.36 and 91% positive correlations (Fig. 1d). To determine whether transcriptional activity associates with the protein–RNA correlation of differentially expressed genes, we plotted the RNA abundance distributions for all samples in bins of protein–RNA correlations. This showed that RNA abundance was not associated with protein–RNA correlation (Fig. 1e). Likewise, the gene-wise correlation analysis showed that 617 genes and 795 instances (including the same genes but different pairwise comparisons) had significant changes in the RNA and protein levels. Gene-wise correlation showed 86.4% concordant changes in protein and RNA (Spearman correlation = 0.63; Fig. 1f). This suggests that differentially expressed genes have high correlation between the RNA and protein levels.

### Accessible chromatin peaks inform protein–RNA correlation

**Fig. 2:**
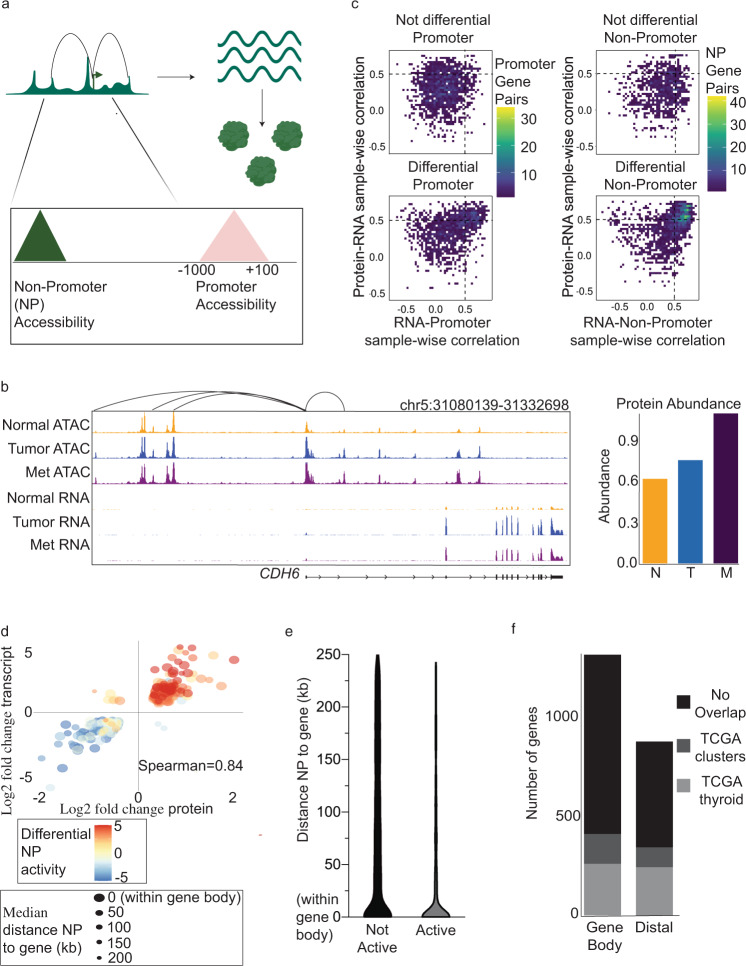
Analysis of ATAC-seq peaks association with RNA and protein abundances. **a** Schematic of peaks linked to genes. **b** Visualization of peak–gene links for *CDH6* and gene expression of *CDH6*. Sample size; normal ATAC = 28, tumor ATAC = 30, met ATAC = 35, normal RNA = 27, tumor RNA = 30, met RNA = 30, normal protein = 21, tumor protein = 22, and met protein = 22. **c** Density of sample-wise correlation values, RNA–promoter and protein–RNA (left) and RNA–non-promoter and protein–RNA (right). The top graph includes only not differential regulatory elements (promoters on left and non-promoters on right) and the bottom graph includes only differential regulatory elements (promoters on left and non-promoters on right). **d** Gene-wise correlation for genes with differential non-promoter activity. **e** Distance between non-promoters to genes for active genes vs genes with no differential activity. **f** Overlap between TCGA ATAC-seq clusters and differential non-promoters within gene body (left) and distal to gene targets (right).

Many approaches have shown that accessible chromatin acts in *cis* to regulate transcription^9,18,19^. Utilizing the genome-wide data of chromatin accessibility and RNA abundances, we quantified the regulatory effect of a comprehensive set of loci on transcription. A promoter or non-promoter (NP) element can have an activating effect, which can be predicted with correlation values^20^. We used an expression-based algorithm to link ATAC-seq peaks, which represent accessible chromatin, to their expected gene targets. We defined peaks into two categories, promoter regulatory elements (−1000 and +100 of its gene’s TSS) and NP regulatory elements (Fig. 2a)^8^. A NP element may be assigned to its nearest gene or linked to a gene that skips over several other genes. Our method of peak gene linkage identified likely gene targets by testing correlation of RNA to the *cis*-ATAC-seq peaks against a null-model for each gene (see “Methods” for details). Figure 2b provides an example of peak gene linkages for *CDH6* and also the associated RNA and protein expression. Overall, we identified 66,000 peak gene linkages (Supplementary Fig. 1f).

Previous research has shown that promoter accessibility is largely invariable while NP elements change in induced systems and between cell types^21^. We examined how regulatory elements associate with the landscape of differential expression in this metastatic thyroid cancer cohort. Figure 2c shows the distribution of sample-wise correlations for promoter elements only (left graphs) and NP elements (right graphs). The *y*-axis of the graphs is the protein–RNA sample-wise correlation. The *x*-axis is the sample-wise correlation of chromatin accessibility of regulatory elements and RNA abundance of their gene targets. The regulatory elements that are graphed are linked to differentially expressed genes at either measure of expression (i.e., RNA and/or protein). The region of interest on the graphs is the upper-right quadrant as these gene-regulatory element pairs show high paired correlation values (defined as correlation values >0.5, which represents an upper bound in the data). Differential promoter elements showed significantly more high paired correlation values compared to not differential promoter elements (18.4 vs 4.3%, respectively, Fig. 2c). Furthermore, we conducted linear regression of the protein levels based on the RNA abundance and regulatory element accessibility. The model identifies that genes with differential RNA expression predicts protein levels better than genes with only differential protein expression, which supports that the chromatin association with protein levels is mediated through transcription (Supplementary Fig. 2c, d). In addition, there is significantly more protein predictability of genes with differential promoters compared to not differential protomers (protein predictive significance = 8.19e−22, see “Methods”). The right graphs show that genes with differential NPs enrich for highly paired correlation values (42.7 vs 7.5% high paired correlation values for differential NPs vs not differential NPs, Fig. 2c). In addition, differential NPs significantly predict protein levels compared to not differential NPs (protein predictive significance = 7.50e−15, Supplementary Fig. 2a). The analytical framework was formatted into an R package that can utilize new datasets as well (https://github.com/asanghi7/epigenoproteomics).

Given the enrichment of high paired correlation values in differential NPs and the predictive nature of differential NPs for protein levels, we further investigated the relationship between differential NPs and protein levels. Across all pairwise comparisons, we identified 202 differentially expressed genes that are significantly changed at the RNA and protein levels and associate with differential NP (Fig. 2d). Gene-wise correlation for these genes is higher than gene-wise correlation for all genes (Spearman = 0.84, 96% concordant directions of log fold changes vs Fig. 1f, Spearman = 0.64, 86.6% concordant directions). The differential NP activity score, which summarizes the total log fold change of the dynamic elements (see “Methods”), matches with the directionality of the log fold change of gene expression. Also, the majority of the linked differential NP peaks reside proximal to the gene. Lastly, the number of gene NPs was largely composed of comparisons between tumor vs normal (715 pairs) and met vs normal (1115 pairs), with very few met vs tumor comparisons (14 pairs).

Attributes such as distance between NP elements and their gene targets can distinguish how chromatin accessibility regulates expression^22,23^. NP elements are known to act upon their gene targets at variable distances, ranging from several megabases away to within the transcriptional unit. Evidence suggests that elements contained within the gene body may be more likely to regulate expression than elements distal from the gene^24–26^. Therefore, we assessed the genetic distance (in kilobases) of NP elements to the gene body of differentially regulated genes. The differentially expressed genes at RNA and protein levels map to proximal differential NPs (Active category on Fig. 2e) compared to not differential NPs that target not differentially expressed genes (Not active on Fig. 2e). The violin plot shows that the distance distribution for these active NP is significantly enriched for activity within the gene body compared to the NP linked to genes with no dynamic activity (Fisher’s exact test = 3.71e−69). We find that, within the active subset, the NPs contained within the gene body have more protein predictive significance compared to the protein predictive significance of NPs outside the gene body (Fisher’s exact test = 3.61e−05).

Much evidence shows that regulatory elements are cell-type specific, and thus, it is expected that regulatory elements in this cohort would overlap with independent data on thyroid-specific regulatory landscapes^26,27^. The Cancer Genome Atlas (TCGA) profiled the chromatin accessibility of 32 tumor types and identified 18 clusters of regulatory landscapes; one cluster is the thyroid cluster. Comparison of our active NPs with those of the TCGA’s thyroid cancer ATAC clusters (Fig. 2f) revealed that 27.5% of NPs within the gene body and 19.8% of elements distal to the gene body overlap with TCGA peaks. These percentages are not expected according to random overlap with TCGA thyroid elements because the genome distance covered within the gene body is less than or equal to the genome distance analyzed in the distal region that is upstream to the gene. Furthermore, the number of active gene body-localized NPs overlapped significantly more with TCGA thyroid peaks compared to active distal NP elements (Fisher’s exact test = 1.24e−14, see “Methods”). Therefore, the gene body-localized regulatory elements are more likely to be identified in external datasets, implying a more important regulatory function compared to distal elements.

### Gene body chromatin accessibility is associated with protein–RNA correlation

**Fig. 3:**
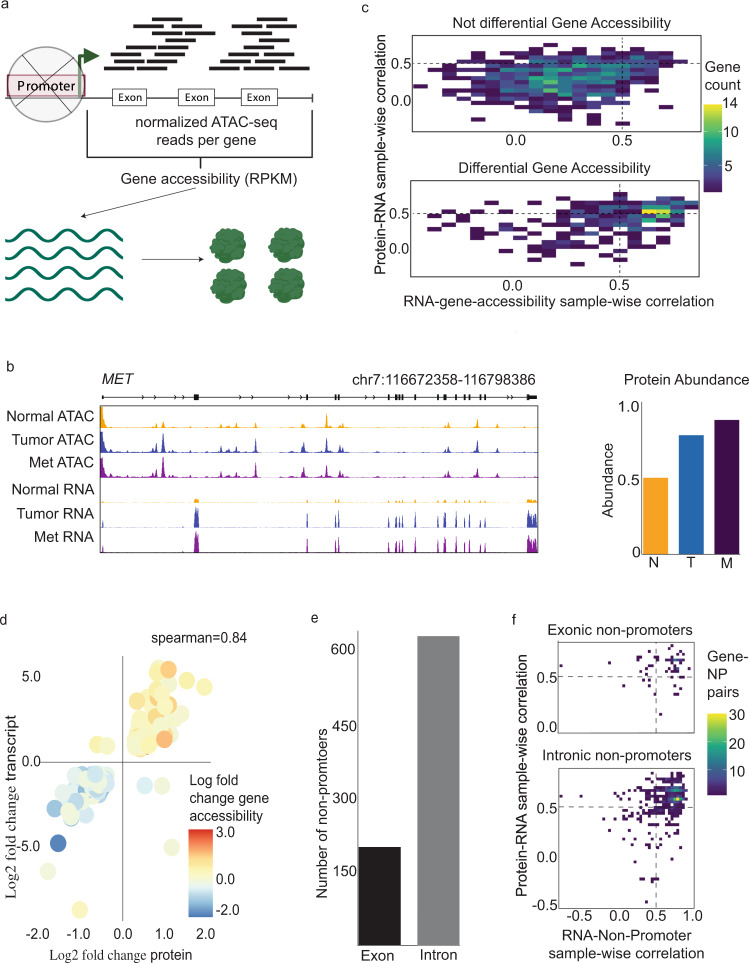
Association of gene accessibility with RNA and protein abundances. **a** Schematic of the gene accessibility metric. **b** Visualization of gene accessibility and RNA and protein abundances for *MET* gene (top) and *FN1* gene (bottom). Sample size; normal ATAC = 28, tumor ATAC = 30, met ATAC = 35, normal RNA = 27, tumor RNA = 30, met RNA = 30, normal protein = 21, tumor protein = 22, and met protein = 22. **c** Density of co-correlation between gene accessibility and gene expression. **d** Gene-wise correlation for genes with differential gene accessibility. **e** Frequency of exonic non-promoters and intronic non-promoters for differentially accessible genes (left) and distribution of the ratio of exonic length to intronic length for differentially accessible genes (right). **f** Comparison of exonic non-promoters (top) vs intronic non-promoters’ sample-wise correlation (bottom).

We found that regions of NP accessibility contained within the gene body are associated with coordinated transcription and translation. Although typically peaks are used to identify important chromatin features, we also tested whether dynamic accessibility across the gene body associated with changes in gene expression. To measure cumulative gene accessibility, we utilized a measure employed for RNA sequencing (RNA-seq): normalized reads per kilobase million (RPKM) (Fig. 3a), which was quantified per gene for each sample. RPKM values were then used in sample-wise and gene-wise correlations similar to the analysis in Fig. 2. As a visual example of how accessibility appears across the gene, we show signal tracks for the *MET* gene and its corresponding RNA and protein levels (Fig. 3b). Chromatin accessibility profiles of *MET* show that chromatin accessibility is clustered within peaks.

Across the landscape of all 5384 proteins detected, we measure the gene accessibility correlation with RNA abundance and protein abundance, using the same distribution of sample-wise correlations as in Fig. 2c. The top subpanel shows the distribution of sample-wise correlation for genes with no differential gene accessibility in any pairwise comparison (Fig. 3c). In contrast, differential accessibility in differentially expressed genes (bottom subpanel) enriches for co-correlated genes (23.0 vs 2.1% high paired correlation values for differentially accessible genes vs not differentially accessible genes; protein predictive significance = 6.551e–09, Supplementary Fig. 2b).

Gene accessibility integrates several pieces of information that relate to gene expression such as DNA regulatory elements and chromatin remodeling as a consequence of RNA polymerase elongation^28^. Given that gene accessibility contains information about the activity of the gene, we expected that the differential changes in accessibility will match with differential changes in expression. As shown in Fig. 3d, gene-wise correlation for the subset of genes with differential gene accessibility has a high correlation of protein and RNA log fold changes (Spearman = 0.84; 211 genes; 98% concordant log fold changes in expression). In addition, the gene accessibility matched the directionality of the log fold changes of expression (Fig. 3d). These results concur with the result in Fig. 2d, in which the three layers of data showed similarly matched directionality.

The results so far suggest that gene body accessibility (RPKM) behaves similarly to the NP peaks. To further assess the regulatory information contained in gene bodies, we investigated whether regulatory elements contained within these active gene bodies enriched for exonic or intronic NP elements. The expectation based on epigenetic studies is that regulatory elements follow a distribution based on the length distribution such that a NP element is more likely to be found in an intron than at exon at the rate of the ratio of intronic length to exonic length in the genome. Thus, we enumerated the number of active exonic and intronic NP contained within differentially accessible genes (Fig. 3e). For these 124 genes, the mean exonic length fraction of the gene body length is 0.11, meaning the intronic regions are on average 9 times longer than exonic regions. The number of active exonic NP elements is 126 and the number of active intronic NPs is 622 (Fig. 3e). Thus, the number exonic active NP elements are marginally more enriched than the expected density (binomial test *p* = 1.48e−06)

Both exonic and intronic NP regulatory elements have been shown to act as enhancer regions^29^. Indeed, we found the sample-wise correlation and protein predictive significance support that exonic and intronic NPs have similar function (Fig. 3f). The high paired correlation values for exonic vs intronic NPs were similar (75 vs 72%), and the protein predictive significance was marginally better in exonic NPs (*p* value = 3e−4). This supports that differential accessibility within the gene body corresponds to active regulatory elements contained within the gene body and these elements have protein predictive power. Overall, we observe that gene body regulatory elements have strong positive correlations with protein levels.

### Mitogen-activated protein kinase (MAPK) transcription factors (TFs) drive protein–RNA correlation in thyroid cancer

**Fig. 4:**
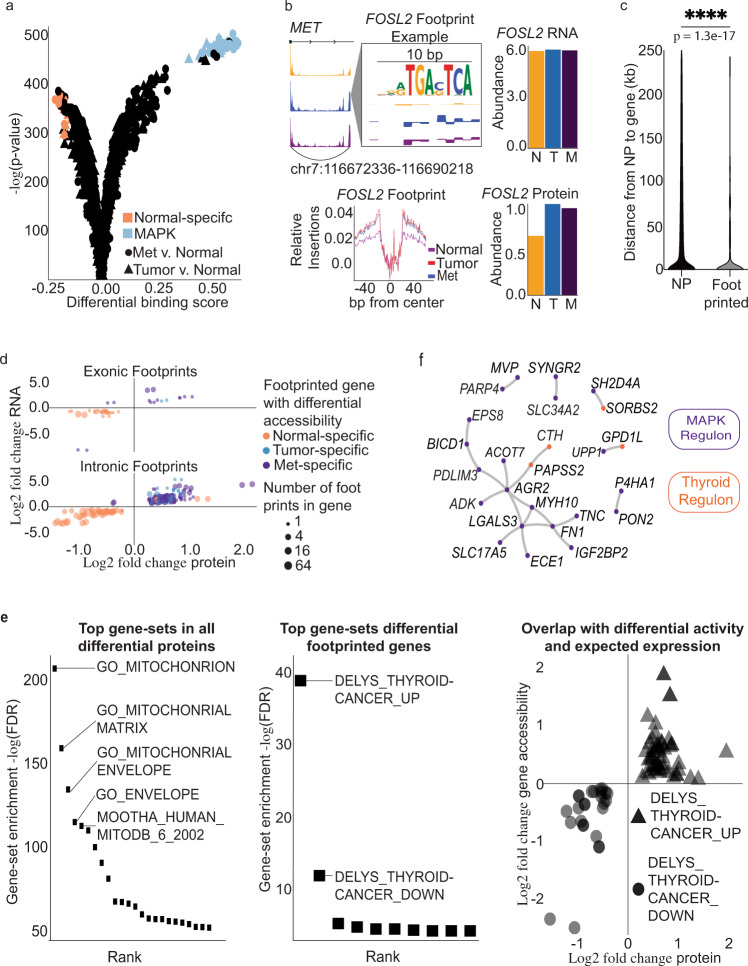
Transcription factor activity in the gene body coordinates RNA and protein expression. **a** Differential transcription factor footprints between tumor/met and normal. **b** Visualization of a FOSL2, a MAPK transcription factor, footprint within the MET gene body (top left). Comparison of genome-wide transcription factor footprints of FOSL2 between normal, tumor, and met (bottom left). Comparison of RNA expression of FOSL2 between normal, tumor, and met (top right). Comparison of protein expression of FOSL2 between normal, tumor, and met (bottom right). **c** Distance distribution of footprinted non-promoters linked to differential expression compared to distance between not active non-promoters to their gene target. Statistical test was two-sided Fisher’s exact test, comparing the number of gene body non-promoters (NP) vs distal NP (*p* = 1.3e−17). **d** Comparison of gene-wise correlation for genes with exonic vs intronic differential footprints. **e** Functional significance of activity based on gene sets. Left graph shows the top gene sets enriched in the differential protein subset of genes. The middle graph describes the top gene sets of footprinted differentially active genes. The left graph shows that footprinted differentially active genes that overlap with known thyroid cancer gene sets have expected changes in protein and gene accessibility between tumor/met vs normal. **f** Protein–protein interaction network of tissue-type-specific regulons.

Regulatory elements mediate gene expression via TF binding. Within putative functional chromatin-accessible regions, footprints of TF binding can be detected^30,31^. We conducted differential TF footprinting in the ATAC-Seq peaks to identify genome-wide bound motifs with greatest difference in predicted binding between every pairwise comparison. First, tumor and metastases had near identical differential footprints when compared to normal. Figure 4a shows the most enriched motifs in tumor and met compared to normal. Given that thyroid cancer is a MAPK-driven tumor, we expected the tumors to harbor active TFs in the MAPK pathway bindin^11,32^. Indeed, we identified a family of MAPK motifs (*FOS*, *JUN*, *JDP*, *BATF*) enriched in tumors and mets, compared to normal. In contrast, we expect that normal thyroid will enrich for lineage factors, and we observe thyroid transcription factor 2 (*TTF-2*, also known as forkhead box protein *FOXE1*)^33^, a thyroid-specific TF essential for thyroid development, is differentially bound in normal compared to tumors/mets. Overall, the top most motifs enriched in normal thyroid are *FOXE1*, *NFATC2*, *NFATC3*, *RFX2*, *RFX5*, *TEAD3*, and *TEAD4*. Several of these TFs bind to consensus DNA motifs, and our prediction method is insensitive to this similarity of motifs. Even though several loci are predicted to have several TFs bound, this does not interfere with the aim to show the specific genes throughout the genome that are controlled by the same regulon set. In tumor and met, the top differential TF footprints are *FOSL1JUNB*, *JUNvar.2*, *FOSL1JUN*, *FOSL2JUNB*, *FOSJUN*, *FOSJUND*, *Smad2Smad3*, *FOSBJUNB*, *FOSJUNB*, *JUNB*, *JUND*, *FOS*, *FOSL1JUND*, *BATF*, *FOSL2JUND*, *FOSL2JUN*, *JUNJUNB*, *FOSL1*, *JDP2*, *BATFJUN*, *BATF3*, and *FOSL2* (Fig. 4a).

Given that a set of TFs are most differentially bound across the genome, we conditioned active loci on whether there is evidence of TF binding in a state-specific manner. Thus, to identify differential footprints at specific loci, we searched for evidence of state-specific TF binding in one state (e.g., Tumor or Met) and no evidence of TF binding in the opposite state (e.g., Normal). We show the genome track of *MET* (gene) layered within information about differential footprints within the gene body (Fig. 4b). There is one site within an intron that contained the *FOS2L* motif and showed active TF binding (top-right graph Fig. 4b). Furthermore, genome wide, we saw an enrichment of footprints in tumor and met compared to normal (bottom-left subpanel of Fig. 4b). This genome-wide footprinting analysis suggests that TFs in the MAPK pathway are actively bound significantly more in tumor and met than in normal.

We expect that the predicted TFs footprinted are expressed in the samples. The bar graphs show the expression at the *FOSL2* RNA level (top right Fig. 4b) and protein level (bottom right Fig. 4b). As expected, *FOSL2* is expressed in tumor and met at both the RNA and protein levels. However, the differential activity cannot be explained entirely by differential expression. We find that the RNA levels are similar between tumor/met compared to normal. However, the protein level of *FOSL2* is significantly higher in tumor and met compared to normal (FDR < 0.1). This not only suggests that post-translational regulation may affect activity of *FOSL2* in thyroid cancer but also indicates that our footprinting approach likely detects functional TF–chromatin interactions.

Regulatory elements within the gene body likely mediate function via differential TF binding, and this function results in gene expression transcriptionally and ultimately translationally. We tested this hypothesis on differentially expressed genes at the protein and RNA levels. We show that the distribution of differential footprints within NPs was significantly shifted toward locations within the gene body as compared to the distribution of distances of all NPs linked to differential RNA (Fig. 4c). We choose differential transcripts’ NPs as the null because this captures the general distribution of distances between peak to gene links (Fisher’s exact test *p* value = 1.3e−17; Fig. 4c). We found that differentially footprinted gene body NPs significantly predict protein abundance compared to non-differential NPs (protein predictive significance = 3.1e−26, Supplementary Fig. 2e). As a positive control for transcription regulation, we demonstrated that proximal enhancers regulate the expression of long noncoding RNA (Supplementary Fig. 3). This suggests that regulatory information proximal to the gene is crucial for coordinating transcription and translation.

Given that NPs are more common in intronic vs exonic regions, we expected to observe a similar trend with differential footprinting. Based on the mean direction of differential gene accessibility, we identified matched differential footprints within the gene body. Figure 4d delineates by color that the differential footprint almost entirely matches the mean direction of expression change at the protein and RNA levels. Interestingly, metastases had several more differential footprints compared to tumors (51 vs 18), suggesting that metastases have more induced changes than tumors. Furthermore, we show that intronic footprints make up the large majority of differential footprints in the gene set with differential accessibility. Overall, 88 out of 161 differentially accessible genes were significantly enriched relative to the number of not differentially accessible genes with any differential footprints in NP regions (Fisher’s test *p* value = 3.6e−07). Therefore, the gene body-accessible chromatin properties associated with high protein–RNA correlation also extend to TF binding.

Differential gene accessibility could have a predicted biological impact if the regulated protein targets interact with one another in gene networks. We observed that gene body accessibility and regulatory activity selected for gene networks relevant to thyroid cancer (Fig. 4e). We found that the top gene sets enriched in all differential proteins are non-specific and likely not relevant to thyroid cancer biology. These top gene sets are generic mitochondrial and metabolic pathways (left graph Fig. 4e). However, when we specifically examined the differentially expressed proteins that contain active footprints within their gene body, then the most enriched gene sets are known thyroid cancer genes (middle graph Fig. 4e). Furthermore, the genes contained within these thyroid cancer gene sets had expected protein expression and gene accessibility as the genes upregulated in thyroid cancer had increased gene accessibility and protein expression in tumor and met compared to normal. Similarly, genes downregulated had decreased accessibility and protein expression (left graph Fig. 4e). Given that these gene sets are largely derived from transcript microarray data, this is orthogonal validation that integrated epigenomics and proteomics identifies cancer-type-specific gene sets.

### Integrated epigeno-proteomics identifies cancer gene regulons

Much information about dysregulated genes in cancer has been historically derived from transcriptome data. For example, *ERK*-driven tumors, which encompass a large fraction of cancer types, including thyroid cancer, are clustered based on expression of specific genes from microarray data^34,35^. Although proteomics is gaining traction in cancer, many tumor types have yet to be profiled with shotgun proteomic technologies. From our integration of epigenomics and proteomics, we find a subset of highly regulated and coordinated differentially expressed proteins in the progression of thyroid cancer. We used these proteins to build a protein–protein interaction subnetwork, focusing on nodes that are modulated by differential regulons between tumor and metastases compared to normal (permuted *p* value = 9.96e−06) (Fig. 4f)^36^. We found that many proteins under regulation of thyroid-specific MAPK TFs are predicted to interact in tumors. Several of the listed genes are known to be involved in thyroid cancer progression (*FN1*, *LGALS3*, and *TNC*)^34,37^. Our network adds connected genes that also have coordinated flow of information from gene accessibility and differential footprints to gene expression. This set of proteins potentially form regulons that are essential for thyroid cancer. Furthermore, we identified several interacting genes that are downregulated in thyroid tumors and mets that are under regulation of the normal thyroid regulon (Fig. 4f).

### Application of integrated epigeno-proteomics in breast cancer (BRCA) subtypes

**Fig. 5:**
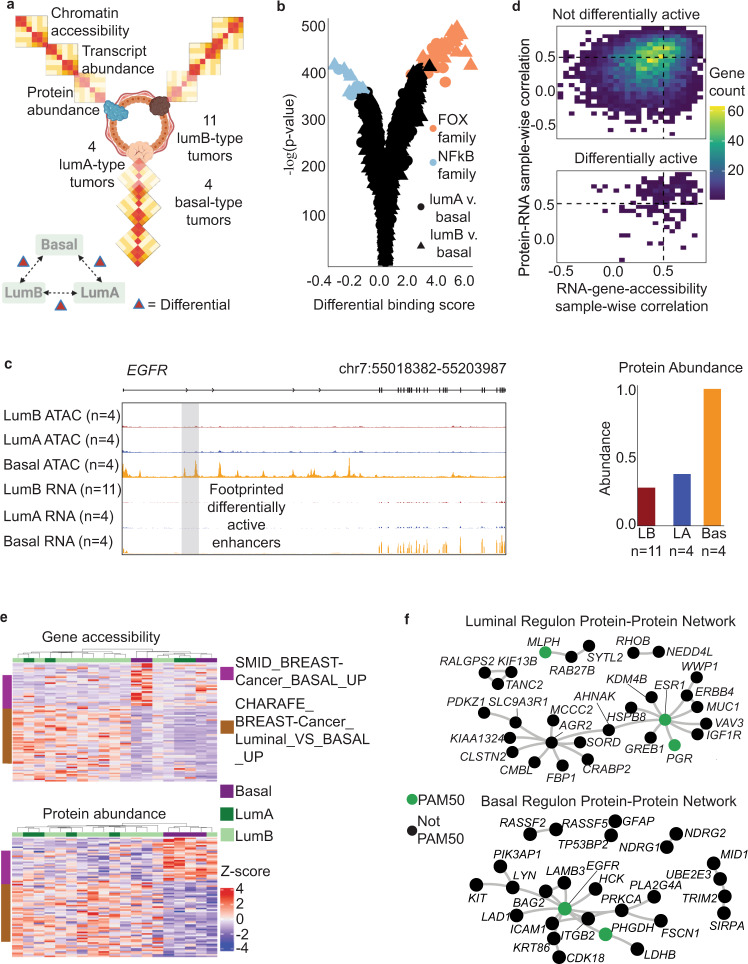
Application of analytical framework to breast cancer cohort. **a** Cohort characteristics, includes 19 breast cancer (BRCA) samples (11 luminal B, 4 luminal A, and 4 basal). **b** Differential transcription factor footprints between luminal A/B and basal subtypes. **c** Visualization of gene accessibility and transcript and protein abundances for *EGFR* in BRCA subtypes. Biologically independent tissue chunks from each disease group (i.e., lumA, lumB, and basal) are averaged to produce the signal plots. The gray highlighted region on the signal track indicates footprinted differentially active enhancers **d** Density of sample-wise correlation between gene accessibility and expression for not differentially active genes (top) compared to differential active genes (bottom). **e** Heatmaps of gene accessibility (top) and protein abundance (bottom) for genes that are differentially expressed and contain gene body enhancers. Annotation bars delineate samples’ subtypes (top bar) and gene membership in known breast cancer gene sets (left bar). **f** Protein–protein interaction networks for luminal regulons (top) and basal regulon (bottom). Green dots mark known PAM50 genes.

Our results indicate that the landscape of open chromatin associates with protein–RNA correlation in thyroid cancer. To examine whether this is the case for other cancers, we analyzed the TCGA BRCA cohort, which has the largest set of samples that are jointly profiled for chromatin accessibility, RNA abundance, and protein abundance (Fig. 5a). However, TCGA did not profile normal breast samples for epigenomic and proteomic information, making tumor–normal differential calling impossible. Instead, we performed differential omics analysis between the BRCA PAM50 molecular subtypes, which in this case includes luminal A, luminal B, and basal. These three subtypes are based on a 50-gene RNA abundance classifier called PAM50^10^. Because these molecular subtypes harbor unique histological and clinical properties, knowledge of differential regulons between them could inform clinical care.

TCGA conducted ATAC-seq on 78 BRCA samples and used the corresponding RNA-seq data from these samples to assign ATAC-seq peaks to genes. Furthermore, iTRAQ-labeled proteomics was conducted on 22 out of the 78 samples in the CPTAC project, and their method detected 6120 proteins in all the samples. We used our analytical framework on these 22 samples to validate whether gene body enhancers coordinate gene expression in BRCA subtypes.

Figure 5b shows that the luminal subtypes of BRCA distinguish themselves from basal subtype based on their TF footprints. Luminal A/B tumors have a higher level of FOX activity (*FOXB1*, *FOXC1*, *FOXA3*, *FOXA2*, *FOXA1*, *FOXP1*, *FOXD2*, *FOXO3*, *FOXF2*, *FOXC2*, *Foxj3*, *FOXE1*, *FOXK2*, *Foxl2*, *Foxf1*, *FOXD1*, *FOXO6*, *FOXL1*, *FOXP3*, *FOXO4*, *FOXI1*, *FOXK1*, *FOXG1*, *FOXP2*, *Foxj2*, *FOXN3*, *GRHL1*)*.* Basal tumors have a higher level of nuclear factor-kB activity (*REL*, *RELA*, *RELB*, *SPIC*, *NFKB1*, *NFKB2*, *SOX4*, *SOX6*).

These observations suggest that footprints contained within the gene body of differentially active genes can be used to distinguish BRCA subtypes. An example is shown for the *EGFR* locus, which is known to be important in the basal subtype (Fig. 5c)^38^. Contained within the *EGFR* locus were basal-specific *REL* footprints (highlighted in Fig. 5c), and these footprints associated with basal-specific gene expression at the RNA and protein levels. These footprinted enhancers have previously been linked to gene expression of *EGFR*^8^.

Our hypothesis is that BRCA subtypes would utilize a similar regulatory mechanism as we have shown in thyroid cancer progression. Since we have identified specific TFs that distinguish BRCA luminal and basal types, we test whether gene body accessibility and transcript abundance predict protein levels for genes that harbor the expected TF-binding sites within the gene body of their targets. Using a similar linear model statistical test, we find that active footprints contained within the gene body enriched for high paired correlation between accessibility and expression (44.6 vs 8.9% high paired correlation values for differentially active genes vs not differentially active genes; modified protein predictive significance = 4.23e−19; Fig. 5d).

Using the subset of genes with predicted protein levels based on transcription and differential footprint activity contained within the gene body, we display heatmaps of the gene accessibility and protein abundance for each sample (Fig. 5e). The top figure shows that gene accessibility clusters approximately according to subtype (basal and luminal subtypes). Furthermore, the genes detected in our analysis overlapped with known BRCA gene sets as shown in the row annotations. The bottom heatmap shows that protein expression clustered basal and luminal subtypes. Similar to gene accessibility, gene expression clustered according to known BRCA gene sets.

We applied our methodology to identify regulons that differentiate basal and luminal subtypes (Fig. 5f). Using differential proteins associated with subtype-specific footprints, we build a protein–protein interaction network that is centered around known PAM50 genes (Fig. 5f: green dots) (permuted *p* value for luminal network = 1.86e−07 and for basal network = 6.04e−06). The luminal network includes 19 genes, not used in PAM50, that are connected to the PAM50 genes. The basal network includes 15 genes that are connected to 2 known PAM50 genes that define the basal subtype. Therefore, integrated epigeno-proteomics could potentially be used to identify important protein–RNA regulons that distinguish biological subsets in cancers.

The gene body contains important regulatory information that distinguishes BRCA subtypes and thyroid cancer progression. The gene body enhancers, although proximal to gene, operate further distances than promoters and are expected to interact within the chromatin looping architecture. We analyzed whether predicted gene body enhancers in our data landed within conserved contact domains across human cell types. Using the published ENCODE ChIA-PET data, we identified that 24 out of 25 genes (excluding IGF2BP2) in the metastatic thyroid cancer gene network (Fig. 4f) harbored gene body enhancers within conserved domains^3^. Likewise, 56 out of 56 genes in the BRCA subtype gene networks had gene body enhancers within conserved contact domains. Gene body enhancers are likely in contact with their upstream promoter, because, across the proteome, 82% of all genes have their entire gene body contained within these conserved contact domains.

## Discussion

We report an integrated epigeno-proteomic dataset from metastatic thyroid cancers based on chromatin accessibility, transcriptomics, and proteomics. Our utilization of normal thyroid tissue for these omics assays allowed us to derive tumor-specific regulatory principles. In particular, we find that chromatin structure can be used to predict highly correlated protein–RNA regulons. We identify that the most likely location of an enhancer site associated with differential gene expression is within the gene body of its target, which is unexpected given the preponderance of studies that emphasized chromatin looping from distal enhancers as a key mode of transcriptional regulation. Importantly, previous studies have been based solely on transcriptional readouts, which is distinguished from our study that integrates RNA and protein data.

Our integrative analytical framework results in a model in which proximal enhancers are associated with coordinated transcription and translation of differentially expressed genes in cancer. These enhancers putatively bind their state-specific TFs, which likely directly or indirectly associated with the coordinated expression. Although enhancers contained within the transcription unit have been identified, it is not known whether these enhancers are necessary for driving coordinated transcription and translation. In fact, it is plausible that the accessible regions define key genes whose expression reflects rapid and direct response to protein levels independent of other regulatory mechanisms. We speculate that, in rapidly dividing cells, direct response to gene regulation at proximal enhancers is subjected to less regulation downstream and thus protein levels more directly correlated with both gene expression and chromatin accessibility. Nonetheless, this approach allowed us to identify protein–RNA regulons that likely distinguish important disease groups.

We observe these effects in two scenarios: (1) progression from normal thyroid to metastatic thyroid cancer and (2) divergent development of three molecular subtypes of BRCA. We identify dynamic enhancers in thyroid cancer tumors that putatively drive thyroid cancer programs as shown in our functional analysis of these gene sets. These gene sets connect to our existing knowledge of the mutation-based drivers of disease, particularly MAPK activation. Thyroid cancer, much like many other cancers, requires better diagnostics and therapeutics that target metastatic disease, and the set of genes that have coordinated regulation and expression likely are targets that should be tested for clinical validity.

For the second scenario, we identify highly regulated and coordinated genes in PAM50 molecular subtypes of BRCA. Molecular characteristics of these tumors are based on transcriptome studies, which may inherently miss molecular information related to phenotype. Our integrative approach isolates active gene regulation that likely drives transcriptional output and results in translational output. We identify a protein–protein interaction that expands the known PAM50 genes and known gene sets assigned to basal and luminal subtypes and shows that our method can connect genes to known molecular features. This study raises hypotheses about the molecular features that define cancers and the mechanisms that coordinate the induced phenotypes.

The clinical impact of this study alludes to a clearer map of regulatory features that connect gene expression to tumor phenotype. In this study, we present a model that identifies important cancer genes that have correlated RNA and protein within an epigenetic context which supports that they are actively induced in the cancer system. This prioritizes the gene targets that determine the tumor phenotype and also offers epigenetic targets that may be leveraged as precision therapeutic targets. Furthermore, as we move to more molecular-based stratifications of tumor types, we envision that our model will be important for determining the phenotypes of aggressive tumors and also subtypes that are not determined by cancer mutation drivers.

Ultimately, this study reinforces that the intronic information within the transcriptional unit coordinates the gene’s expression. Our model uses evidence of co-correlation between chromatin regulatory elements to RNA and protein and evidence that accessibility matches with RNA and protein abundances. The evidence advocates for a model in which enhancer elements within the gene body actively regulate gene expression, which ultimately leads to coordinated transcription and translation. This study informs the relatively understudied field of how the global chromatin landscape regulates protein expression especially in the context of cancer phenotypes that arise from malignant progression and divergence into molecular subtypes.

## Methods

### Thyroid cancer cohort selection

This study was approved by the Institutional Review Board of Stanford University (IRB-11402). Informed consent was obtained prior to enrollment of all subjects. The study was conducted according to the principles of the Declaration of Helsinki (2008). Patients had confirmed diagnosis of papillary thyroid carcinoma. At the time of surgery, tissues were harvested and flash-frozen and stored at −80 °C.

### Sample characteristics

Our collection involves a range of patients with mostly first diagnosis (*n* = 33) of thyroid cancer and a few recurrence cases (*n* = 3). For all first-diagnosis cases, we collected adjacent normal thyroid, the primary tumor, and one to three cervical lymph nodes metastasis. These samples were processed and cut by a trained pathologist. Samples were collected from 36 patients, in total, including 35 metastatic neck lymph node tumors, 30 primary tumors, and 28 adjacent normal thyroids. Based on histological diagnoses, 32 cases were papillary thyroid carcinoma, 2 were follicular thyroid carcinoma, and 2 were poorly differentiated thyroid carcinoma.

### ATAC-seq sample and library preparation

Each tissue sample was cut from a larger tissue chunk on dry ice to aliquot 10 mg pieces for ATAC-seq preparation and nuclei were isolated according to published protocols^8^. All of the steps were carried out at 4 °C. A frozen tissue fragment ∼20 mg was placed into a pre-chilled 2-ml Dounce homogenizer containing 2 ml of cold 1× homogenization buffer (320 mM sucrose, 0.1 mM EDTA, 0.1% NP40, 5 mM CaCl_2_, 3 mM Mg(Ac)2, 10 mM Tris pH 7.8, 1× protease inhibitors (Roche, cOmplete), and 167 μM β-mercaptoethanol, in water). Homogenized tissue was transferred to a pre-chilled 2 ml Lo-Bind Eppendorf tube. An equal volume (400 μl) of a 50% iodixanol solution (50% iodixanol in 1× homogenization buffer) was added and mixed by pipetting to make a final concentration of 25% iodixanol. In all, 600 μl of a 30% iodixanol solution (30% iodixanol in 1× homogenization buffer containing 480 mM sucrose) was layered underneath the 25% iodixanol mixture. Another layer of 40% (40% iodixanol in 1× homogenization buffer containing 480 mM sucrose) was pipetted at the bottom of the tube. In a swinging-bucket centrifuge, nuclei were centrifuged for 20 min at 3000 r.c.f. The interface between the 29 and 35% iodixanol solutions was collected as the band of nuclei. Nuclei tagmentation was done with Tn5 transposase and TD buffer from Illumina (cat# FC-121-1030). After reaction clean-up, library fragments were amplified using 1× NEBnext PCR master mix and 1.25 μM of custom Nextera PCR primers 1 and 2 (Supplementary Data 1), using the following PCR conditions: 72 °C for 5 min; 98 °C for 30 s; and thermocycling at 98 °C for 10 s, 63 °C for 30 s, and 72 °C for 1 min. Processing of samples was performed in case batches of 12 samples such that every sample for an individual were included in a batch and with approximately equal numbers of tumors, normals, and metastases in each batch. For each tissue sample, at least 100,000 nuclei were isolated such that 50,000 nuclei replicates could be used to generate technical replicates.

### ATAC-seq library preparation and high-throughput sequencing

After library preparation, library concentration was checked by quantitative PCR using the KAPA Library Quantification Kit. Libraries were sequenced to 50,000–200,000 reads on an Illumina MiSeq Sequencer to check library quality. Library quality was assessed by the TSS enrichment score with a cutoff of 7 was used to determine whether a library was of sufficient quality to deep sequence based on ENCODE performance metrics^39,40^. After quality control, we generated 230 libraries that were pooled together at equal molar concentrations, and this pool was purified on a 6% poly-acrylamide TBE gel (BioRad cat# 4565015) as described in the TCGA ATAC-seq paper to remove excess primers^8^. After purification, each library pool was quantified by Bioanalyzer and sequenced on two lanes of NovaSeq6000 S2 using paired-end 101-bp reads to an average depth of 25 million reads per replicate.

### ATAC-seq data analysis

ATAC-seq FASTQ reads were processed by the ENCODE pipeline for ATAC-seq v1.5.0 (https://github.com/ENCODE-DCC/atac-seq-pipeline). For each sample, peak calling was performed on the Tn5-corrected single-base insertions using the MACS2 callpeak command with parameters–shift −75–extsize 150–nomodel–call-summits–nolambda–keep-dup all -p 0.01. The peak summits were then extended by 100 bp on either side to a final width of 200 bp. From all 230 libraries (2–3 technical replicates per sample), we merge the peak summit regions to create a complete peak set for the thyroid cancer cohort. We identify 611,754 peaks with an average size of 328 base pairs. To obtain the number of independent Tn5 insertions in each peak, each corrected insertion site was counted using bedtools coverage. This was done for all individual technical replicates and 611,754 × 230 counts matrix was compiled. The counts matrix was then normalized by using edgeR’s cpm(matrix, log = TRUE, prior.count = 5) followed by a quantile normalization using preprocessCore’s normalize.quantiles in R. Lastly, we merged technical replicates using the log2 average from the normalized counts matrixs^8^. Differential analysis for peaks between normal, tumor, and met was conducted with limma, and FDR was set to 1% with Benjamini–Hochberg (BH) correction^41^. This FDR was chosen because ATAC-seq is sensitive similar to other next-generation sequencing approaches, but it is subject to noise in using a union peak set to identify differential peaks. A FDR of 0.01 with BH correction would mitigate false positives from our peak analysis strategy.

### Identifying ATAC-seq peak-gene linkage

Given that there is an intractably large space of peaks and genes (611,754 peaks and 28,000 genes) in our experiments, we must reduce the space of potential peaks that regulate their gene targets. We linked ATAC-seq peaks to their genes using a RNA-correlation approach^8,20^. For each gene, we quantify the Pearson correlation of the RNA count’s rlog transformed value from DESeq2 with every peak in the pan-thyroid cancer peak set that is within 500 kb, 250 kb in each direction, of the gene’s first TSS^42^. We calculated correlations separately for each tissue type (normal, tumor, and met). The correlation is tested against a null model, which is the gene expression with 10,000 random trans peaks (i.e., peaks on other chromosomes) that are expressed in the thyroid cancer cohort, and this distribution of the correlations with random peaks is used to construct a Gaussian null model, which was used to calculate *p* values. Random peaks are a reasonable approach because peak values do not confound correlation values. Like the experimental correlation, the null model is generated for each tissue type. Since the null model is calculated for each gene, it controls for any bias contained within the gene and its expression. We set our FDR to 0.05 with BH correction to identify the correlated peaks to genes. This FDR was chosen based on prior studies in cancer data and optimization based on our samples^8^. By subsampling our data, we identified that setting FDR to 0.05 results in a similar number of peak-gene links across the tissue types (i.e., normal, tumor, met) (see below). In all, 96% of all peaks assigned to genes lie within conserved contact domains^3^.

### ATAC-seq peak visualization

We merged all the insertion sites across all samples and replicates for each tissue type and visualize a summary of ATAC-seq by tissue type. To do this, the genome was binned into 100-bp intervals using the bedtools genome function^43^. Windows and number of insertions per tissue type were counted using bedtools coverage. The coverage was normalized to the total number of insertions. The normalized data were converted to bigwigs. All track figures in this paper show groups of tracks with matched *y*-axis scales.

### Gene accessibility metrics

We develop metrics to measure the chromatin accessibility across variable-sized regions defined by the transcription units. To measure gene accessibility, we count the ATAC-seq reads across the gene excluding all promoter regions identified from our peak analysis. The counts are divided by the gene length in kilobases and by number of reads in the sample per million, which results in an RPKM. Across the cohort and all gene accessibility RPKMs, we quantile normalize using preprocessCore’s normalize.quantiles in R.

### TF footprinting

We merged all the bam files across all samples and replicates for each tissue type and subsampled the reads to 350 million reads per tissue type. These three bams (i.e., normal, tumor, and met) were processed by TOBIAS, which conducts TF footprinting analysis from ATAC-seq reads^30^. Peaks were called using macs:–nomodel–shift -75–extsize 150–broad; broad setting was used to better capture the regions with footprints. We utilized the eighth release (2020) of JASPAR position frequency matrices to identify TF motifs^44^.

### RNA-Seq sample preparation and high-throughput sequencing

Each tissue sample was cut from a larger tissue chunk on dry ice to aliquot 10 mg pieces for RNA-seq preparation. We extracted RNA using the AllPrep DNA/RNA Mini Kit (cat#: 80204). RNA sample RINs were confirmed to be >7 by Bioanalyzer. Libraries were prepared using Illumina TruSeq Stranded Total RNA LP Gold and IDT-TruSeq RNA UD Idx (cat# 20020598 and 20020598). The libraries were prepared in house on the semi-automatic Agilent Bravo NGS in a single batch. We pooled all 88 libraries at equal ng concentrations and sequenced the pooled library on the NovaSeq6000 S2 using paired-end 101-bp reads with compatible primers (Supplementary Data 1). On average, we achieve 45 million reads per sample. Differential analysis was conducted with DESEQ2 in R and FDR was set to 10% (BH correction)^42^. This FDR was chosen based on prior RNA-seq experiments, which show a good sensitivity from the next-generation sequencing approach, but the optimal FDR also accounts for false positives due to normalization^42^.

### RNA-seq data analysis

Reads were mapped to hg38 genome, and GENCODE version 32 was used to annotate genes. Reads were mapped using default parameters from STAR^45^. From the mapped reads, transcripts were counted at a gene level using HTSeq used to enumerate the number of reads per sample^46^. Transcript counts per million was used in downstream analysis. Differential analysis between tissue types was conducted with DEseq2 in R and FDR was set to 10% (BH correction)^42^.

### RNA-seq data visualization

Reads from BAM files of samples from the same tissue type were merged together and then subsampled to 200 million reads. The subsampled reads were converted to big wigs for visualization using deeptools bamCoverage^47^.

### Proteomics sample preparation

Each tissue sample was cut from a larger tissue chunk on dry ice to aliquot 20 mg pieces for proteomics. Tissue samples were disrupted using bead beating and sonication in lysis buffer (6 M guanidine, 10 mM TCEP, 40 mM CAA, 100 mM Tris pH 8.5). The supernatant was collected and heated at 95 °C for 5 min. After protein reduction and alkylated, protein concentration was measured using the BCA Kit (ThermoFisher). Protein extract was cleaned up by acetone precipitation at −20 °C overnight. The protein pellet was washed with acetone three times and air-dried. The pellet was resuspended in 6 M guanidine and 100 mg was digested using LysC (1:100 protease to protein ratio) for 2 h followed by trypsin (1:50) digestion overnight at 37 °C. Peptides were cleaned up using Waters HLB column and subsequently labeled using TMT11 Plex (ThermoFisher) in 100 mM TEAB buffer. An equal amount of protein from each tissue were pooled together as a reference sample. Tissue samples were randomized such that all samples from the same patient were in the same batch and equal number of normal, tumor, and met were in each batch (three samples per tissue type) and equal amount of them and one common reference sample and a GTEx-thyroid reference was multiplexed into one sample. To ensure equal mix, we mixed a small amount of each sample first and adjusted the amount of each sample for the final run based on the mass spectrometry results of the small mix. Each sample was run in multiple batches with different TMT labels and in different runs with other samples.

About 15 µg of multiplexed sample was loaded to Waters 2D LC system for online fractionation. Peptides were separated by reverse-phase chromatography at high pH in the first dimension, followed by an orthogonal separation at low pH in the second dimension. In the first dimension, the mobile phases were buffer A: 20 mM ammonium formate at pH 10 and buffer B: acetonitrile. Peptides were separated on an Xbridge 300 µm × 5 cm C18 5.0 µm column (Waters) using 12 discontinuous step gradient at 2 µl/min. In the second dimension, peptides were loaded to an in-house packed 75 µm ID/15 µm tip ID × 25 cm Sepax GP-C18 1.8 µm resin column with buffer A (0.1% formic acid in water). Peptides were separated with a linear gradient from 5 to 30% buffer B (0.1% formic acid in acetonitrile) at a flow rate of 300 nl/min in 180 min. The LC system was directly coupled in-line with an Orbitrap Fusion (Thermo Fisher Scientific).

### Mass spectrometry data acquisition

The Orbitrap Fusion was operated in a data-dependent mode for both MS2 and MS3. MS1 scan was acquired in the Orbitrap mass analyzer with resolution 120,000 at *m*/*z* 400. Top speed instrument method was used for MS2 and MS3. For MS2, the isolation width was set at 0.7 Da and isolated precursors were fragmented by collision-induced dissociation (CID) at a normalized collision energy (NCE) of 35% and analyzed in the ion trap using turbo scan. Following the acquisition of each MS2 spectrum, a synchronous precursor selection (SPS) MS3 scan was collected on the top five most intense ions in the MS2 spectrum. SPS-MS3 precursors were fragmented by higher-energy CID at an NCE of 65% and analyzed using the Orbitrap at a resolution of 60,000.

We used SEQUEST in ProteomeDiscoverer v2.1 (ThermoFisher Scientific) for protein identification. Raw files from 12 fractions of each sample were combined together for a single search against GENCODE V28 human proteome database. Mass tolerance of 10 p.p.m. was used for precursor ion and 0.6 Dalton for fragment ions. The search included cysteine carbamidomethylation as a fixed modification. Peptide N-terminal and lysine TMT 11plex modification, protein N-terminal acetylation, and methionine oxidation were set as variable modifications. Up to two missed cleavages were allowed for trypsin digestion. The peptide FDR was set as <1% using Percolator. For protein identification, at least one unique peptide with a minimum six amino acid length was required. For protein quantitation, only unique peptides with reporter ion mass tolerance of <10 p.p.m. were used. Peptide precursor ion isolation purity should be >50%, signal-to-noise (S/N) >15, and the summed S/N of all channels >200. Peptides passing these criteria were summed, thereby giving more weight to the most intense peptides. We also pooled together all the spectra in this study for a single search at protein FDR of 1% as used in prior mass spectrometry that profiled primary tissue^48^.

### Mass spectrometry data analysis

Protein abundance of each sample was first rescaled so that the total peptide abundance in each channel was the same as the total sum abundance of the reference channels in the same run. In each 11plex sample, if a peptide abundance was missing in a channel, its abundance was set to the minimum value in that run. We report protein abundance at the gene level and as the ratio of the sample channel to the reference channel. To weigh ratio toward peptides with high intensities, which are expected to be more accurate, the unique peptides assigned to a gene were summed in a sample channel and reference channel and then the ratio was calculated. In total, we identified protein abundances from 10,408 genes. Overall, 5418 genes were detected in all samples and used in downstream analysis. Protein abundances were normalized to the reference channel abundances in each run, making relative abundances comparable across batches. Relative protein abundances were log2 transformed and then averaged across technical replicates. There were a total of 65 samples used in differential analysis (21 Normal thyroids, 22 Tumors, and 22 Mets). Differential analysis between tissue types was conducted with limma (FDR = 10%)^49^. This FDR was appropriate for mass spectrometry measurements as they profile a smaller set of genes and have a smaller dynamic range compared to next-generation sequencing.

### Clustering of multi-omics dataset

We performed analysis to cluster the samples based on similarity of each omic data. To visualize the similarity, we utilized *t*-Distributed Stochastic Neighbor Embedding (*t*-SNE) using Rtsne(perplexity = 11, max_iter=10000, pca = TRUE) on each omic data set. We included all genes that were detected across all the omic platforms (5384 genes) and all samples that were profiled across all the platforms (54 samples).

### Proteomics data visualization

Relative protein abundances (not log2 transformed) across tissue types were plotted as medians on bar graphs in R.

### Differential NP activity score

In gene-wise correlation analysis, we develop a metric to assess the total log fold change for all differential NPs assigned to a gene. This metric is called the differential NP activity score. The score is a summation of all the log fold changes of that peak for a specific pairwise comparison. For example, the differential NP activity score for FN1 gene for the tumor vs normal comparison would be a summation of all differential NPs’ log fold changes in the tumor vs normal comparison.

### Protein predictive significance test

In the co-correlation analysis, gene-regulatory element pairs are categorized into four categories: (a) no differential activity (at both gene expression and regulatory element), (b) differential gene expression only, (c) differential regulatory element only, and (d) all differential (at both gene expression and regulatory element). From these categories, the significant enrichment of co-correlation was assessed by splitting the samples into two random sets to control for selection bias. One set was used to assess the fraction of co-correlated gene-regulatory element pairs. The second set was tested in a linear model ProteinExpression ~ RNAexpression + RNAexpression × Accessibility of element. This results in a *F*-statistic, and a significance was set at FDR = 0.05, which was chosen to have an appropriately strong test to mitigate false positives. We then assigned accessibility features and gene pairs to functionally relevant categories such as differential gene expression and differential accessibility. The number of pairs below FDR = 0.05 were compared between categories using Fisher’s exact test, which was the protein predictive significance *p* value.

### Epigeno-proteomics R package

The analytical framework of the protein predictive significance test is broadly applicable to matched chromatin accessibility data, RNA expression, and protein expression. The approach utilized three steps to identify genes with protein abundance that correlates with RNA abundance and chromatin accessibility. The first step randomizes the samples to two sets. The second step is to visualize one set of data based on the paired correlation values (RNA–protein and RNA–accessibility Spearman correlations) based on categories of differential features. The third step tests the protein predictive significance, which is used to determine which categories have the most protein predictive significance. This package is publicly available on Github at https://github.com/asanghi7/epigenoproteomics.

### BRCA cohort analysis

We downloaded data from 22 samples of BRCA data (TCGA and CPTAC)^13,50^. From TCGA annotations, we categorized the 22 samples according to their PAM50 molecular subtype (11 luminal B, 4 luminal A, and 4 basal-like, 2 HER2, and 3 unknown). ATAC and RNA were downloaded using TCGA’s SGDC Data Transfer Tool v1.6.0. Raw ATAC-seq data was downloaded with dbGaP approval. ATAC-seq data was reformatted into fastq files with bedtools and then reanalyzed with the ENCODE pipeline for ATAC-seq v1.5.0 (as described above). From these bam files, TF footprinting was conducted using 300 million reads per group (as described above). Gene accessibility quantification was conducted for each sample as described above. Ht-seq-counts RNA counts were downloaded using TCGA’s SGDC Data Transfer Tool. In addition, predicted ATAC-RNA peak-gene links were downloaded from the GDC portal. We downloaded the patient-matched normalized protein abundances from the CPTAC portal, which used iTRAQ-4 labeling proteomics. The differential analyses between the three most abundant subtypes (luminal B, luminal A, and basal) was completed.

### RNA-seq differential analysis

Gene counts downloaded from TCGA were input into DESeq2, which conducted differential analysis at FDR = 0.1 with BH correction^42^. There were a total of 71 samples used in differential analysis (20 basal tumors, 23 luminal A tumors, and 28 luminal B tumors).

### Protein differential analysis

Normalized and transformed data downloaded from CPTAC were input into limma, which conducted differential analysis at FDR = 0.1. There were a total of 62 samples used in differential analysis (18 basal tumors, 21 luminal A tumors, and 23 luminal B tumors).

### Modified protein predictive significance test

For the BRCA data, there were only 22 samples that had matched accessibility, RNA, and protein data. Thus, our statistical test for co-correlation was modified such that we did not split the samples into two datasets as done in the significance test of thyroid cancer data. We categorized the total proteome into two classes: not differentially active and differentially active. Differentially active genes were genes that had differential expression between subtypes in both measures of expression (protein and RNA). In addition, these differentially expressed genes were linked to differential footprints contained within their gene body. For each category, we calculated *F*-statistic *p* value for each gene using the linear model protein ~ RNA + RNA × Accessibility of element. The modified protein predictive significance was calculated by Fisher’s exact test for the number of pairs below FDR = 0.05 in differentially active genes compared to not differentially active set.

### Pathway enrichment

We investigated gene-set enrichments using MSigDB, including their entire collection of gene sets. We uploaded gene sets to the MSigDB website and identified gene sets (FDR = 0.05).

### Protein–protein interaction networks

We used BioGRID 3.5.187 to identify predicted protein–protein interactions. We compared our gene lists with BioGRID data, which identified protein interactions a priori. The networks were loaded in an igraph network on R, and igraph allowed nodes and edges to be trimmed according to their features. The final networks were plotted using ggnetwork in R.

### Permuted *p* value for regulon protein–protein interaction networks

To assess the significance of regulon protein–protein interaction networks (see below), we permuted the BioGRID database that overlapped with the proteome of the specific sample set being used (thyroid or breast). We permuted the 1000 times to generate a null distribution of protein–protein edges and then generated a permuted Gaussian distribution as the null. The *p* value is based on the *z*-score of the observed number of protein–protein links in the experimental protein–protein network.

### Contact domain analysis

Contact domains were based on ENCODE’s ChIA-PET data from 24 cell lines.^3^ They identified 56,893 contact domains that were invariable across the cell lines. Utilizing these conserved domains, we identified regulatory regions that were contained within these contact domains using bedtools intersect.

### Long noncoding RNA analysis

A list of long noncoding RNAs was taken from the hg38 Gencode version 38 release. Using the overlap with all detected genes in our RNA-seq data, we measured the sample-wise correlation of NP accessibility and RNA abundance in peak-gene links. In addition, we assessed the distance of NP peaks to the gene body of their targets.

### Reporting summary

Further information on research design is available in the Nature Research Reporting Summary linked to this article.

## Supplementary information

Supplementary Information

Description of Additional Supplementary Files

Supplementary Data 1

Reporting Summary

## Data Availability

The data that support this study are available from the corresponding author upon reasonable request. The RNA-seq and ATAC-seq data generated in this study have been deposited in the GEO database under accession code GSE162515. The mass spectrometry proteomics data generated in this study has been deposited in the PRIDE database under accession code PXD023078. The raw breast cancer ATAC-seq and RNA-seq data are protected and are not available due to data privacy laws. Access to raw sequencing data from NIH’s dbGaP would require a data transfer agreement. The processed ATAC-seq data are available at the GDC portal (https://gdc.cancer.gov/about-data/publications/ATACseq-AWG). The processed RNA-seq data from matched patients’ samples with ATAC-seq are available in the GDC portal (https://portal.gdc.cancer.gov/projects/TCGA-BRCA). The raw breast cancer proteomics data are available at the CPTAC portal (https://cptac-data-portal.georgetown.edu/study-summary/S029). The processed mass spectrometry data are available in the publication. The predicted protein–protein interactions used in this study are available in the BioGRID database (https://downloads.thebiogrid.org/BioGRID/Release-Archive/BIOGRID-3.5.187). The gene set enrichments used in this study are available in the MSigDB database (https://www.gsea-msigdb.org/gsea/msigdb/). Source data are provided with this paper.

## Source data

Source Data

## Code availability

Integrative analysis code and scripts will be made available upon request. We additionally have created a R package epigeno-proteomics, which uses processed ATAC, RNA, and proteomics data to test protein predictive significance; the package includes sample data from this study. This package is publicly available on Github at https://github.com/asanghi7/epigenoproteomics.
