# Supplementary material for: Chromatin accessibility associates with protein-RNA correlation in human cancer
Source: Nat Commun. 2021 Sep 30;12:5732. doi: 10.1038/s41467-021-25872-1 (PMC8484618; doi:10.1038/s41467-021-25872-1)
Supplement: Supplementary file 1 — Supplementary Information [file 41467_2021_25872_MOESM1_ESM.pdf]

Supplementary Information

Chromatin accessibility associates with protein-RNA correlation in human cancer

Akshay Sanghi[1], Joshua J. Gruber[1,2], Ahmed Metwally[1], Lihua Jiang[1], Warren Reynolds[3], John Sunwoo[4], Lisa Orloff[4], Howard Y. Chang[1,3], Maya Kasowski[1,5,6,7] Michael P. Snyder[1]
1 Department of Genetics, Stanford University, Stanford, California, USA
2 Department of Medicine, Division of Oncology, Stanford University School of Medicine, Stanford, CA
3 Center for Personal Dynamic Regulomes and HHMI, Stanford University
4 Department of Otolaryngology, Division of Head and Neck Surgery, Stanford University School of Medicine, Stanford, CA
5 Department of Pathology, Stanford University School of Medicine, Stanford, CA
6 Department of Medicine, Division of Pulmonary and Critical Care Medicine, Stanford University School of Medicine, Stanford, CA
7 Sean N. Parker Center for Allergy and Asthma Research at Stanford University, Stanford University, Stanford, CA, USA
Correspondence: mpsnyder@stanford.edu

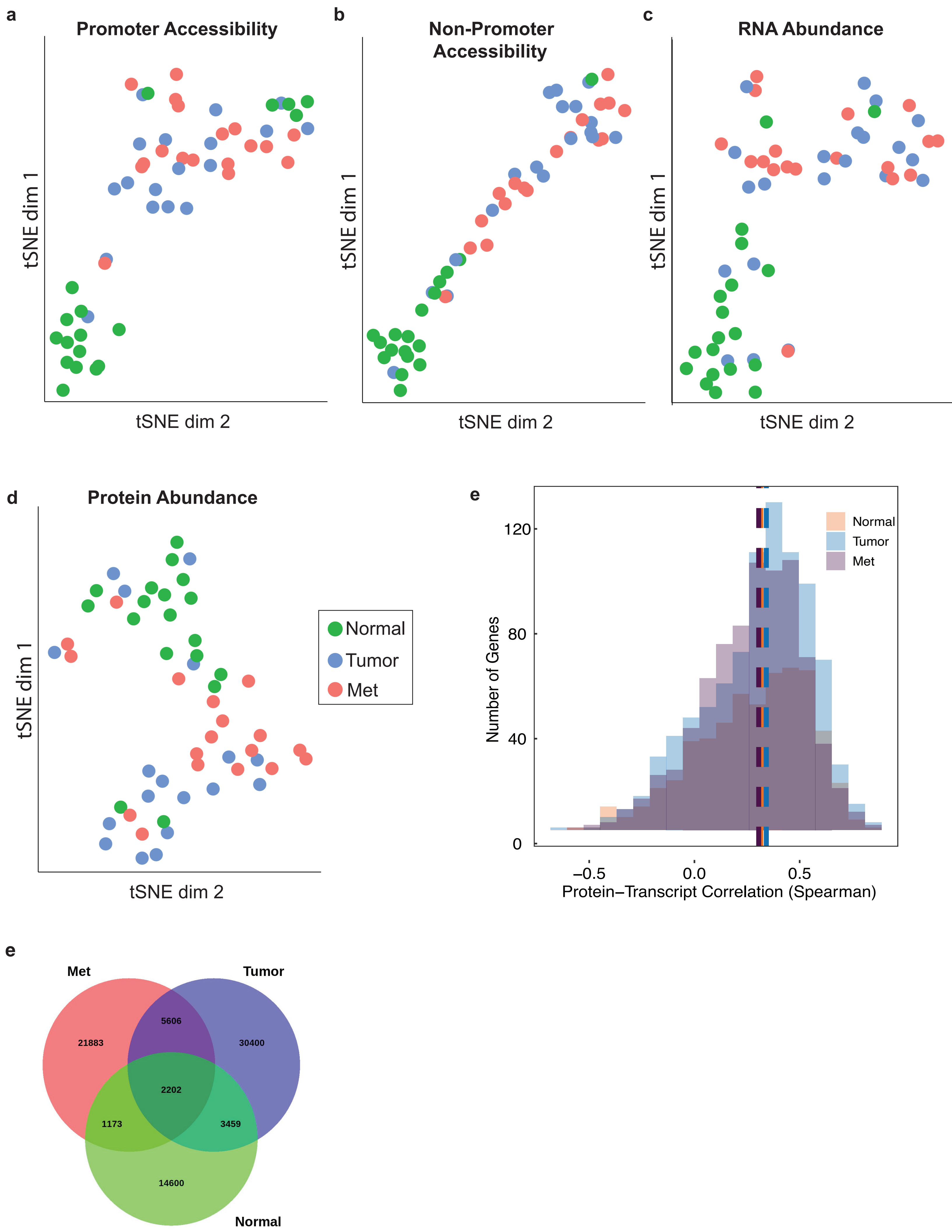
a
Promoter Accessibility
tSNE dim 1
tSNE dim 2
b
Non-Promoter Accessibility
tSNE dim 1
tSNE dim 2
c
RNA Abundance
tSNE dim 1
tSNE dim 2
d
Protein Abundance
tSNE dim 1
tSNE dim 2
Normal
Tumor
Met
e
Number of Genes
0
40
80
120
−0.5
0.0
0.5
Protein−Transcript Correlation (Spearman)
Normal
Tumor
Met
e
Met
Tumor
Normal
21883
5606
30400
2202
1173
3459
14600

Supplementary Figure 1: Heterogeneity of cohort a, tSNE plot of promoter accessibility across all samples. Normal samples are green dots, tumor samples are blue dots, and metastases samples are red dots. **b**, tSNE plot of non-promoter accessibility across all samples. Normal samples are green dots, tumor samples are blue dots, and metastases samples are red dots. **c,** tSNE plot of RNA abundance across all samples. Normal samples are green dots, tumor samples are blue dots, and metastases samples are red dots. **d**, tSNE plot of protein abundance across all samples. Normal samples are green dots, tumor samples are blue dots, and metastases samples are red dots.. **e**, Distribution of sample-wise protein-RNA correlation based on tissue type (i.e. normal, tumor, and metastases). **f**, Venn-diagram of peak-gene links in each tissue type (i.e. normal, tumor, metastases).

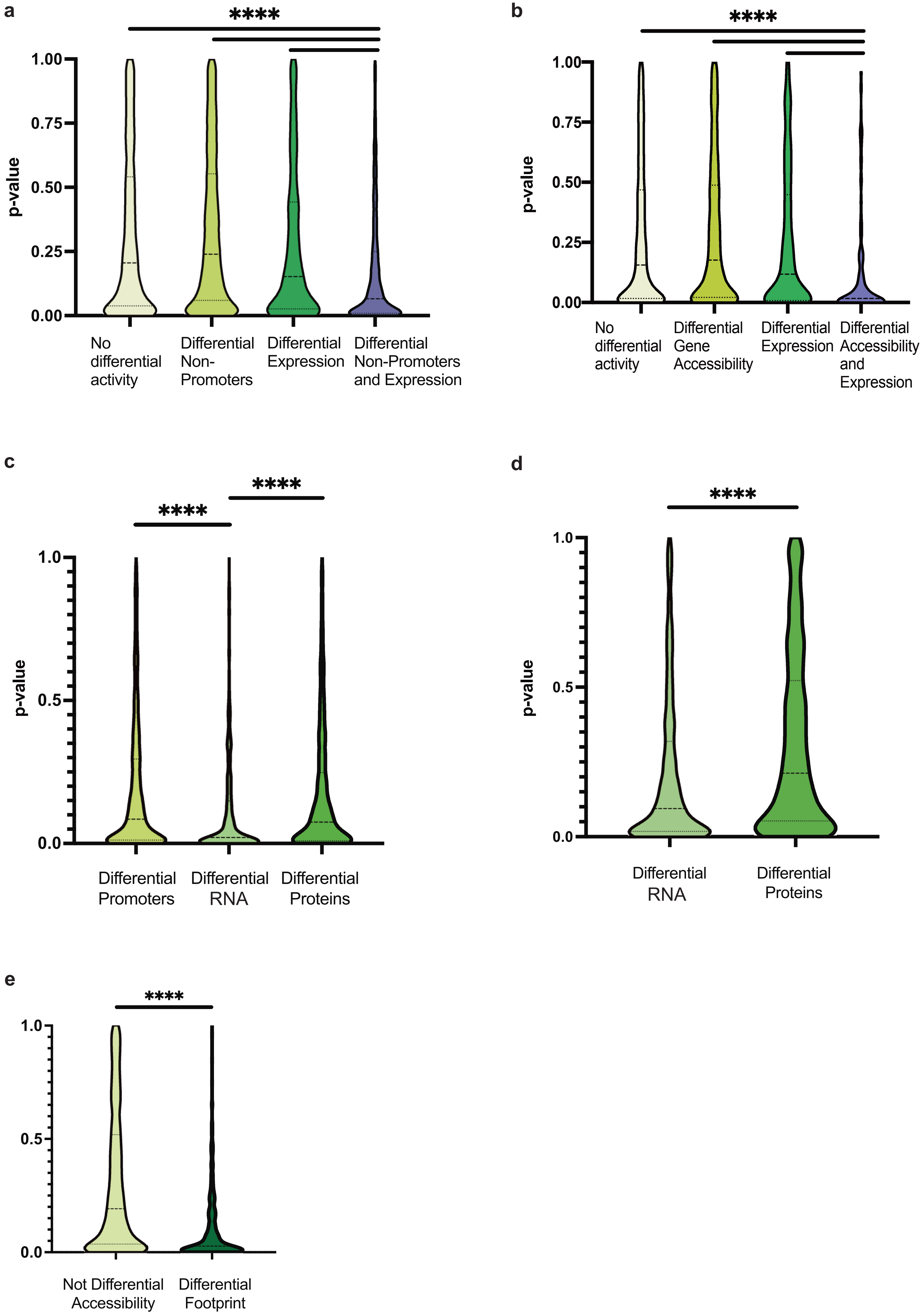
a
****
p-value
1.00
0.75
0.50
0.25
0.00
No differential activity
Differential Non-Promoters
Differential Expression
Differential Non-Promoters and Expression
b
****
p-value
1.00
0.75
0.50
0.25
0.00
No differential activity
Differential Gene Accessibility
Differential Expression
Differential Accessibility and Expression
c
****
****
p-value
1.0
0.5
0.0
Differential Promoters
Differential RNA
Differential Proteins
d
****
p-value
1.0
0.5
0.0
Differential RNA
Differential Proteins
e
****
1.0
0.5
0.0
Not Differential Accessibility
Differential Footprint

Supplementary Figure 2: Protein predictive significance in functional comparisons a, p-values from protein-predictive model for non-promoter-gene links that are 1) not differentially accessible and not differentially expressed (RNA or protein) 2) differentially accessible, and not differentially expressed 3) not differentially accessible, and differentially expressed 4) differentially accessible and differentially expressed. Protein predictive significance test (No differential activity v. Differential Non-Promoters and Expression $p=5.82e-46$; Differential Non-Promoters v. Differential Non-Promoters and Expression $p=5.78e-94$; Differential Expression v. Differential Non-Promoters and Expression $p=8.85e-17$). **b**, p-values from protein-predictive model for genes that are 1) not differentially accessible and not differentially expressed (RNA or protein) 2) are differentially accessible but not differentially expressed 3) not differentially accessible, and differentially expressed 4) differentially accessible and differentially expressed. Protein predictive significance test (No differential activity v. Differential Gene Accessibility and Expression $p=6.39e-17$; Differential Gene Accessibility only v. Differential Gene Accessibility and Expression $p=2.22e-18$; Differential Expression v. Differential Gene Accessibility and Expression $p=6.55e-09$). **c,** p-values from protein predictive model for promoter-gene links that are 1) differentially accessible 2) differentially expressed at RNA level, and not differentially expressed at protein level 3) not differentially expressed at RNA level, and differentially expressed at protein level. Protein predictive significance test (Differential Promoters v. Differential RNA and Expression $p=1.77e-15$; Differential Protein v. Differential RNA $p=3.30e-09$). **d**, p-values from protein predictive model for non-promoter-gene links that are 1) differentially expressed at RNA level, and not differentially expressed at protein level 2) not differentially expressed at RNA level, and differentially expressed at protein level. Protein predictive significance test (Differential RNA v. Differential Protein $p=3.54e-24$). **e**, p-values from protein predictive model for non-promoter-gene links that do 1) not have differential gene accessibility 2) have differential accessibility of gene-bodies that contain non-promoters with tissue-specific transcription factor footprints. Protein predictive significance test (No Differential Accessibility v. Differential Footprints $p=2.11e-25$)

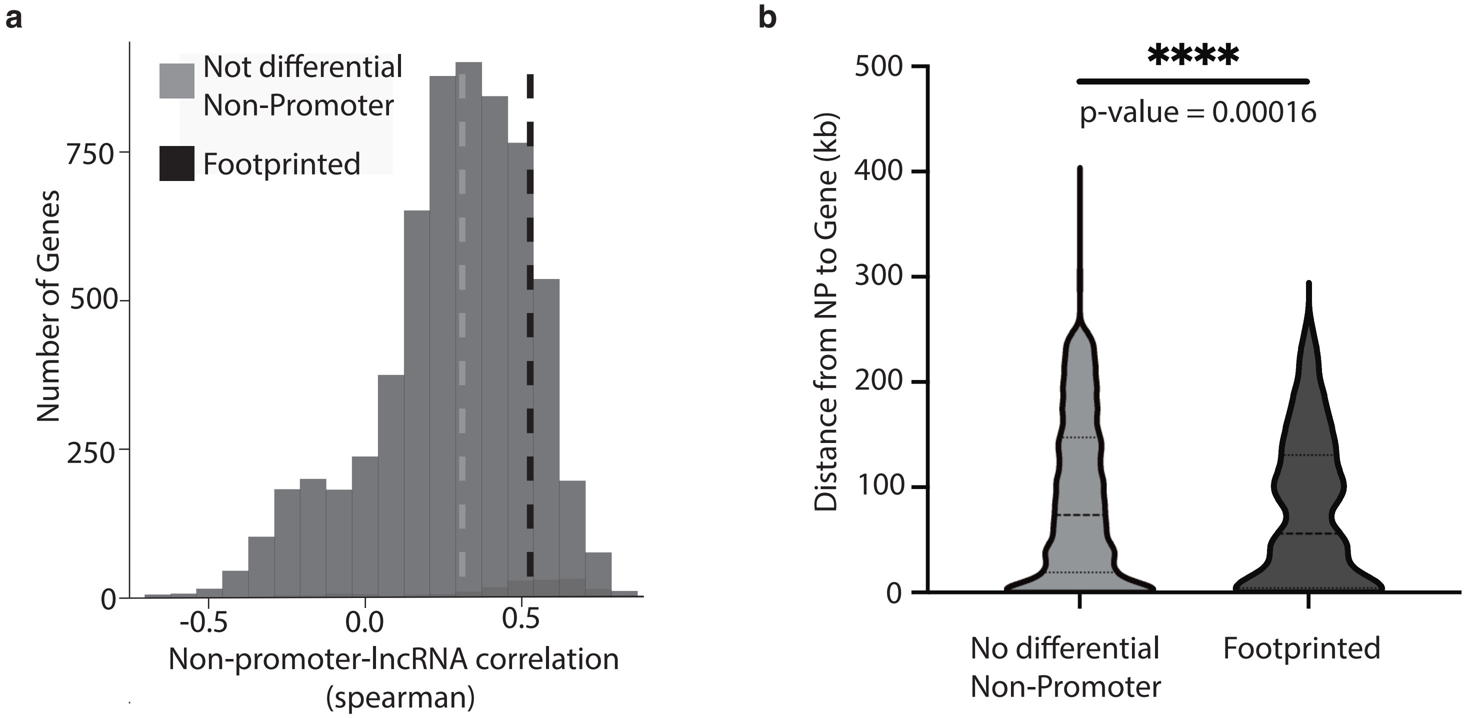
a
Not differential
Non-Promoter
Footprinted
Number of Genes
750
500
250
0
-0.5
0.0
0.5
Non-promoter-lncRNA correlation
(spearman)
b
****
p-value = 0.00016
Distance from NP to Gene (kb)
500
400
300
200
100
0
No differential
Non-Promoter
Footprinted

Supplementary Figure 3: Regulation of long noncoding RNA a, Distribution of non-promoter-RNA correlation (spearman) for gene-non-promoter links that are not differentially accessible and not differentially expressed (gray dotted line marks median correlation) and that are differentially accessible and differentially expressed with tissue-specific footprints in non-promoters (black dotted link marks median correlation). **b**, Distribution of distance of non-promoters to gene links that are not differentially accessible and not differentially expressed (in gray) and that are differentially accessible and differentially expressed with tissue-specific footprints in non-promoters. Statistical test was two-sided fisher's exact test, comparing number of Non-promoters (NP) within gene body v. distal ($p=0.00016$).
