# Supplementary material for: Chromatin accessibility associates with protein-RNA correlation in human cancer
Source: Nat Commun. 2021 Sep 30;12:5732. doi: 10.1038/s41467-021-25872-1 (PMC8484618; doi:10.1038/s41467-021-25872-1)
Supplement: Supplementary file 2 — Description of Additional Supplementary Files [file 41467_2021_25872_MOESM2_ESM.pdf]

Description of Additional Supplementary Files

File Name: Supplementary Data 1
Description: Primer sequences used in ATAC-seq and RNA-seq
