# Supplementary material for: Chromatin accessibility associates with protein-RNA correlation in human cancer
Source: Nat Commun. 2021 Sep 30;12:5732. doi: 10.1038/s41467-021-25872-1 (PMC8484618; doi:10.1038/s41467-021-25872-1)
Supplement: Supplementary file 4 — Reporting Summary [file 41467_2021_25872_MOESM4_ESM.pdf]

nature portfolio

Corresponding author(s): Michael Snyder
Last updated by author(s): Jul 23, 2021

Reporting Summary

Nature Portfolio wishes to improve the reproducibility of the work that we publish. This form provides structure for consistency and transparency in reporting. For further information on Nature Portfolio policies, see our Editorial Policies and the Editorial Policy Checklist.

Statistics

For all statistical analyses, confirm that the following items are present in the figure legend, table legend, main text, or Methods section.

n/a	Confirmed	
☐	☒	The exact sample size (*n*) for each experimental group/condition, given as a discrete number and unit of measurement
☐	☒	A statement on whether measurements were taken from distinct samples or whether the same sample was measured repeatedly
☐	☒	The statistical test(s) used AND whether they are one- or two-sided<br>*Only common tests should be described solely by name; describe more complex techniques in the Methods section.*
☐	☒	A description of all covariates tested
☐	☒	A description of any assumptions or corrections, such as tests of normality and adjustment for multiple comparisons
☐	☒	A full description of the statistical parameters including central tendency (e.g. means) or other basic estimates (e.g. regression coefficient) AND variation (e.g. standard deviation) or associated estimates of uncertainty (e.g. confidence intervals)
☐	☒	For null hypothesis testing, the test statistic (e.g. *F*, *t*, *r*) with confidence intervals, effect sizes, degrees of freedom and *P* value noted<br>*Give P values as exact values whenever suitable.*
☒	☐	For Bayesian analysis, information on the choice of priors and Markov chain Monte Carlo settings
☒	☐	For hierarchical and complex designs, identification of the appropriate level for tests and full reporting of outcomes
☒	☐	Estimates of effect sizes (e.g. Cohen's *d*, Pearson's *r*), indicating how they were calculated

Our web collection on statistics for biologists contains articles on many of the points above.

Software and code

Policy information about availability of computer code

Data collection	SEQUEST in ProteomeDiscoverer v2.1, SGDC Data Transfer Tool v1.6.0
Data analysis	ENCODE ATAC-seq pipeline v1.5.0, TOBIAS v 0.11, Epigenoproteomics v1 (https://github.com/asanghi7/epigenoproteomics), deeptools v3.3.0, bedtools v2.30.0, MSigDB v7.4<br>R v3.6, DESeq2 v1.3, preprocessCore v1.48.0, limma v3.42.2, edgeR v3.28.1, Rtsne v0.15, ggnetwork v0.5.8

For manuscripts utilizing custom algorithms or software that are central to the research but not yet described in published literature, software must be made available to editors and reviewers. We strongly encourage code deposition in a community repository (e.g. GitHub). See the Nature Portfolio guidelines for submitting code & software for further information.

Data

Policy information about availability of data

All manuscripts must include a data availability statement. This statement should provide the following information, where applicable:

- Accession codes, unique identifiers, or web links for publicly available datasets
- A description of any restrictions on data availability
- For clinical datasets or third party data, please ensure that the statement adheres to our policy

The RNA-seq and ATAC-seq data generated in this study have been deposited in the GEO database under accession code #GSE162515 [https://www.ncbi.nlm.nih.gov/geo/query/acc.cgi?acc=GSE162515]. The mass spectrometry proteomics data generated in this study has been deposited in the PRIDE database under accession code PXD023078 [https://www.ebi.ac.uk/pride/archive/projects/PXD023078]. The raw breast cancer ATAC-seq and RNA-seq data are protected and are not available due to data privacy laws. Access to raw sequencing data from NIH's dbGaP would require a data transfer agreement. The processed ATAC-seq data are available at the GDC portal [https://gdc.cancer.gov/about-data/publications/ATACseq-AWG]. The processed RNA-seq data from matched

patients' samples with ATAC-seq are available in the GDC portal [https://portal.gdc.cancer.gov/projects/TCGA-BRCA] The raw breast cancer proteomics data are available at the CPTAC portal [https://cptac-data-portal.georgetown.edu/study-summary/S029]. The processed mass spectrometry data are available in the publication, specifically supplementary table 3, global-proteome-G3 [https://www.ncbi.nlm.nih.gov/pmc/articles/PMC5102256/]. The predicted protein-protein interactions used in this study are available in the BioGRID database [https://downloads.thebiogrid.org/BioGRID/Release-Archive/BIOGRID-3.5.187]. The gene set enrichments used in this study are available in the MSigDB database [https://www.gsea-msigdb.org/gsea/msigdb/]. Source data are provided with this paper.

Field-specific reporting

Please select the one below that is the best fit for your research. If you are not sure, read the appropriate sections before making your selection.

☒ Life sciences ☐ Behavioural & social sciences ☐ Ecological, evolutionary & environmental sciences

For a reference copy of the document with all sections, see nature.com/documents/nr-reporting-summary-flat.pdf

Life sciences study design

All studies must disclose on these points even when the disclosure is negative.

Sample size	36 participants contributed thyroid-cancer tissue specimens. Overall 93 tissue specimens were collected (35 metastatic-neck-lymph-node tumors, 30 primary tumors, and 28 adjacent-normal thyroids). This sample size was set to result in on the order of 20,000 peak-gene links within a disease group similar to published results (Corces et al. 2018 Science). We provided FDR rationales in the Methods section for all analyses. For the breast cancer analysis, 22 samples were used (11 luminal B, 4 luminal A, and 4 basal-like, 2 HER2, and 3 unknown) because these samples had publicly available matched ATAC-seq, RNA-seq, and proteomics data. The breast cancer cohort was sufficient for the curated analysis.
Data exclusions	We excluded 28 thyroid samples from proteomics because the tissues were not compatible with the mass spectrometry machine. We excluded 6 thyroid samples from the RNA-seq dataset because they did not meet sequencing library QC metrics.
Replication	ATAC-seq data had on average 2 technical replicates. All ATAC samples passed the ENCODE guidelines for reproducibility (self-consistency ratio < 2). Proteomics data had at least two technical replicates run of two different mass spectrometers, and the replicates were successful as the average pearson correlation across replicates was 0.70 similar to prior work (Jiang et al. 2020 Cell). Each experimental group (normal thyroid, primary tumors, and metastases) had multiple biological replicates as detailed in the sample size section. These samples were successful replicates as features met strict FDR criteria across various comparisons and the main conclusions were possible with set of biological replicates.
Randomization	Randomization was not relevant to this study. All sequencing methods were conducted such that all samples were pooled together. Proteomic samples were randomized to different runs except all samples from the same patient were run together to support intra-patient comparisons. All samples were used in the study and were assigned to their designated group: normal thyroid, primary tumor, and metastases.
Blinding	Blinding is not relevant to our study - we did not require separate experimental groups.

Reporting for specific materials, systems and methods

We require information from authors about some types of materials, experimental systems and methods used in many studies. Here, indicate whether each material, system or method listed is relevant to your study. If you are not sure if a list item applies to your research, read the appropriate section before selecting a response.

Materials & experimental systems

n/a	Involved in the study	
☒	☐	Antibodies
☒	☐	Eukaryotic cell lines
☒	☐	Palaeontology and archaeology
☒	☐	Animals and other organisms
☐	☒	Human research participants
☒	☐	Clinical data
☒	☐	Dual use research of concern

Methods

n/a	Involved in the study	
☒	☐	ChIP-seq
☒	☐	Flow cytometry
☒	☐	MRI-based neuroimaging

Human research participants

Policy information about studies involving human research participants

Population characteristics	All patients had a histological diagnosis of thyroid cancer. No other clinical variates were used in this analysis.
Recruitment	All patients were recruited under an institutional review board protocol (IRB-11402) at Stanford University. The protocol included language identical to the NIH Genomic Data Sharing Policy. Patients were recruited because they had an aggressive form of thyroid cancer, and were being operated by one surgeon at Stanford. The recruitment strategy reduced bias from inter-surgeon variability, but may introduce bias based on how this study's surgeon operates.
Ethics oversight	Institutional Review Board of Stanford University.

Note that full information on the approval of the study protocol must also be provided in the manuscript.
